# Supplementary figures and images for: Modulation of ADAM17 Levels by Pestiviruses Is Species-Specific
Source: Viruses. 2024 Oct 2;16(10):1564. doi: 10.3390/v16101564 (PMC11512297; doi:10.3390/v16101564)

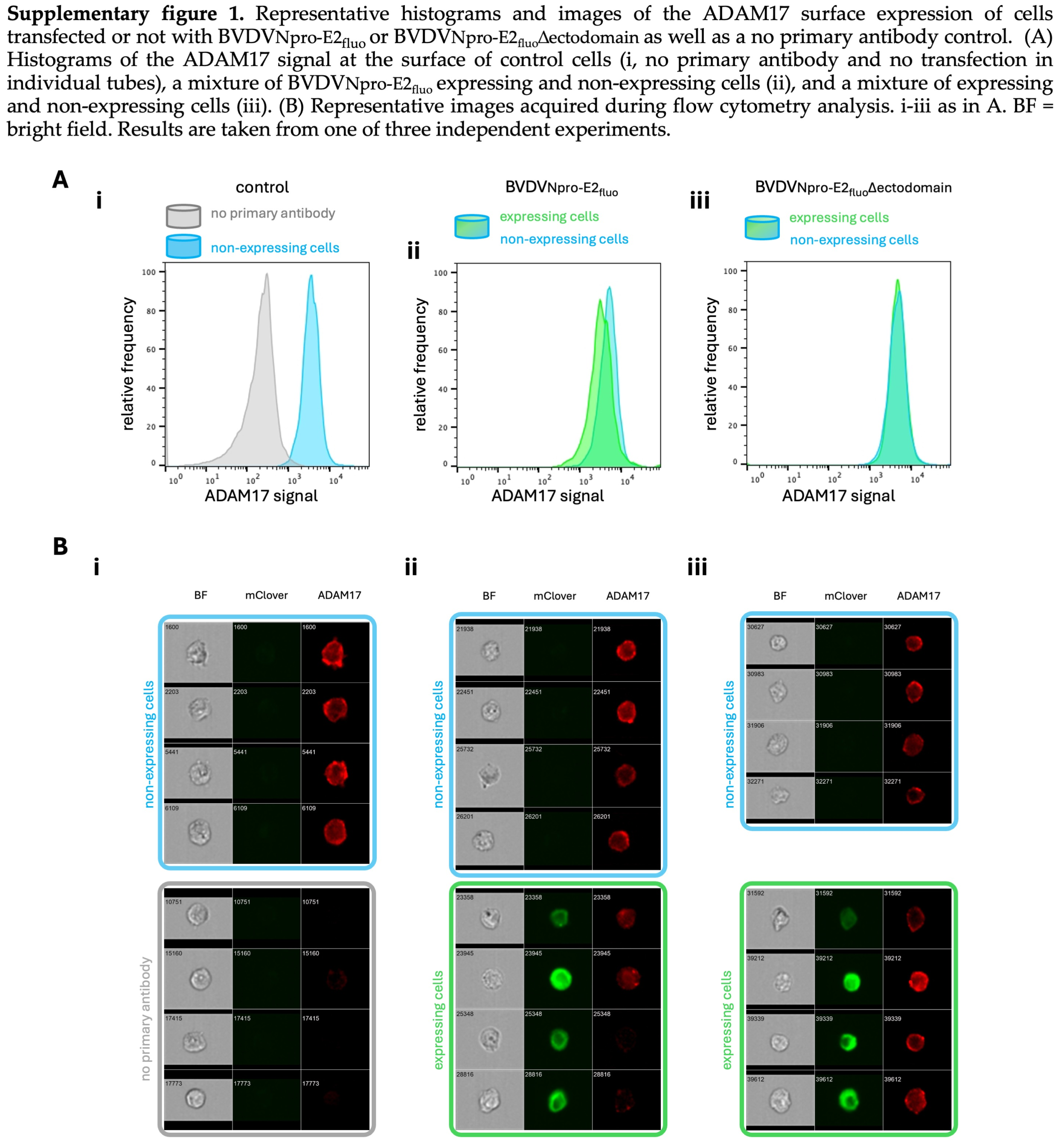

Supplement: Supplementary file 1 [file viruses-16-01564-s001.zip › SupplementaryFigureS1.png]

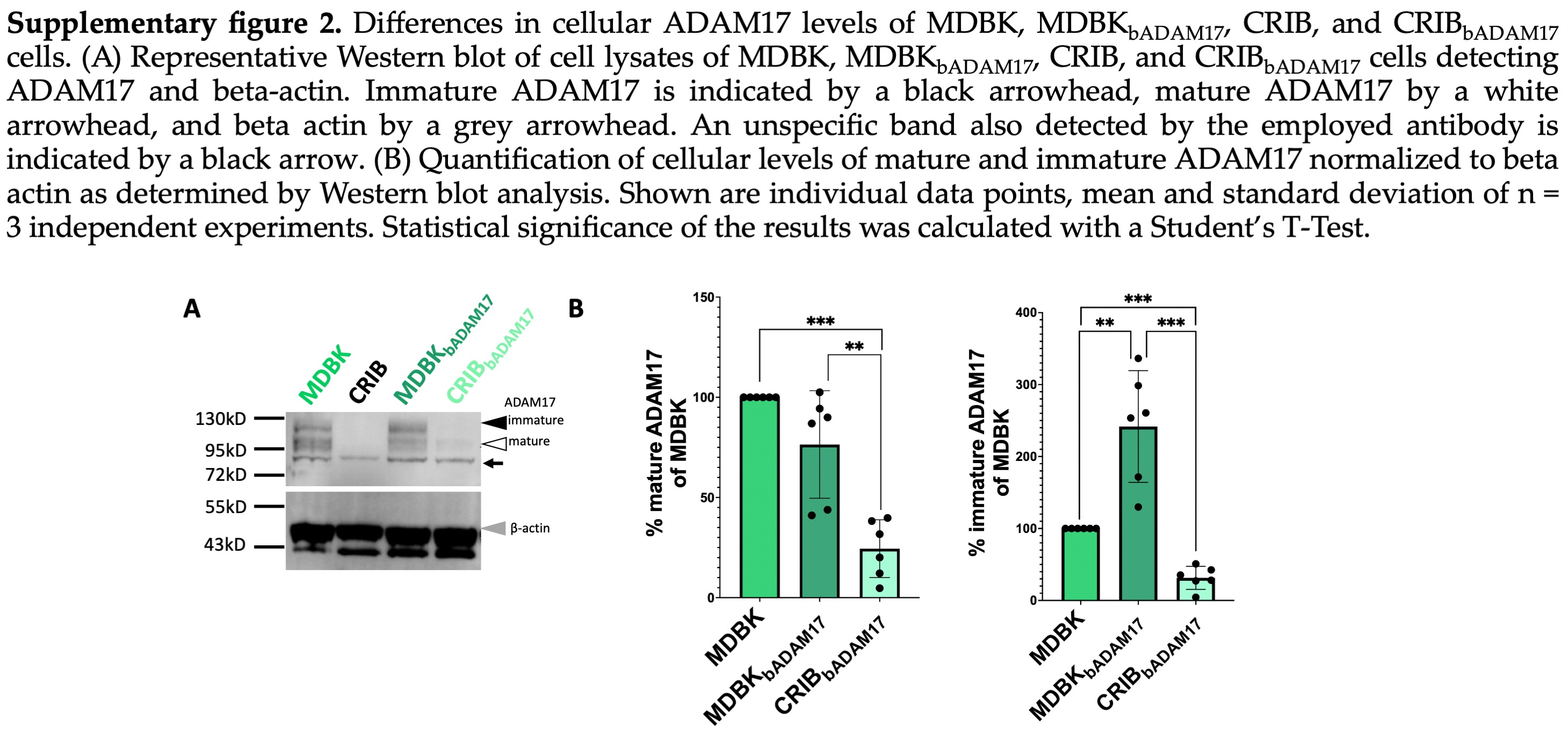

Supplement: Supplementary file 1 [file viruses-16-01564-s001.zip › SupplementaryFigureS2.png]

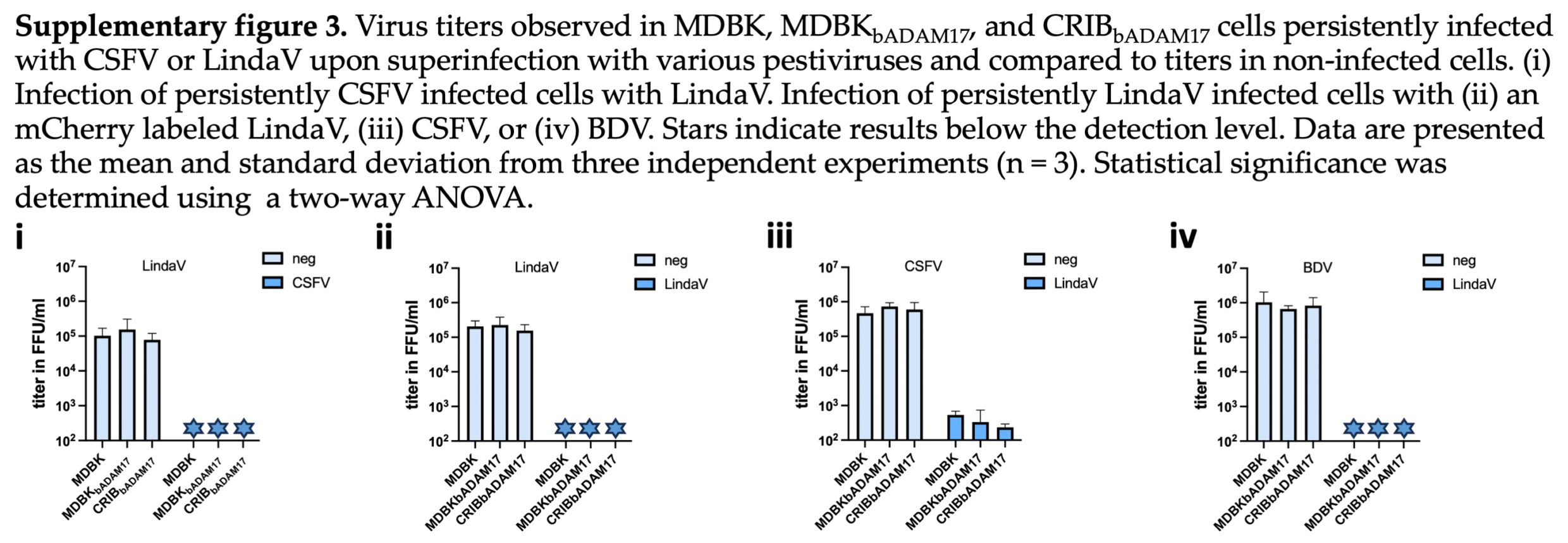

Supplement: Supplementary file 1 [file viruses-16-01564-s001.zip › SupplementaryFigureS3.png]
